# Reconstruction of a Neglected Patellar Tendon Rupture in an Adolescent: A Case Report

**DOI:** 10.7759/cureus.63844

**Published:** 2024-07-04

**Authors:** Filippos Zigras, George A Komnos, Michael Hantes

**Affiliations:** 1 Department of Orthopaedic Surgery & Musculoskeletal Trauma, University General Hospital of Larissa, Larissa, GRC; 2 Department of Orthopaedics, Larissa Hospital, Larissa, GRC

**Keywords:** transpatellar tunnel, hamstring tendon autograft, chronic injury, adolescent, patellar tendon reconstruction, patellar tendon rupture

## Abstract

We present a case of a neglected patellar tendon rupture, misdiagnosed as an anterior cruciate ligament tear, in a 12-year-old child with open physis without an avulsion fracture. The patient was treated with an ipsilateral hamstring tendon autograft with preserved distal insertions, a transpatellar tunnel, and a transtibial fixation. At the final follow-up, the patient had a full range of motion and a fully functional knee. The described technique results in complete muscle strength, full range of motion, and pain-free gait. It can be used in chronic patellar tendon ruptures and is a valuable addition to the therapeutic quiver for this type of injury.

## Introduction

Rupture of the patellar tendon is a relatively uncommon injury and represents 0.6% of musculoskeletal tendinous injuries in the general population, predominantly in patients younger than 40 years of age [1]. In the pediatric population, as the open physis is the weakest part of the muscle-tendon-bone interface during childhood, bony avulsion fractures, such as tibial tubercle or patella sleeve fracture, prevail over tendinous rupture. The incidence of isolated patellar tendon rupture in children, without bony avulsion, is estimated at around 7% of all extensor mechanism injuries [2], and only a few cases are reported in the literature [3-7].

We present a neglected patellar tendon rupture case in a child with an open physis using an ipsilateral semitendinosus and gracilis (STG) tendon autograft. Informed consent was obtained from the patient’s parents, and all clinical images and videos were granted permission for medical publication.

## Case presentation

A 12-year-old boy with no comorbidities presented to our sports medicine outpatient clinic with antalgic pain and an inability to extend his left knee (Video 1). He had experienced a knee injury while playing football four months ago and was initially evaluated elsewhere. The patient was misdiagnosed with an anterior cruciate ligament (ACL) tear and treated with an immobilizer knee brace locked in extension for six weeks.

**Video 1:** Preoperative clinical examination.

**Figure 1:**
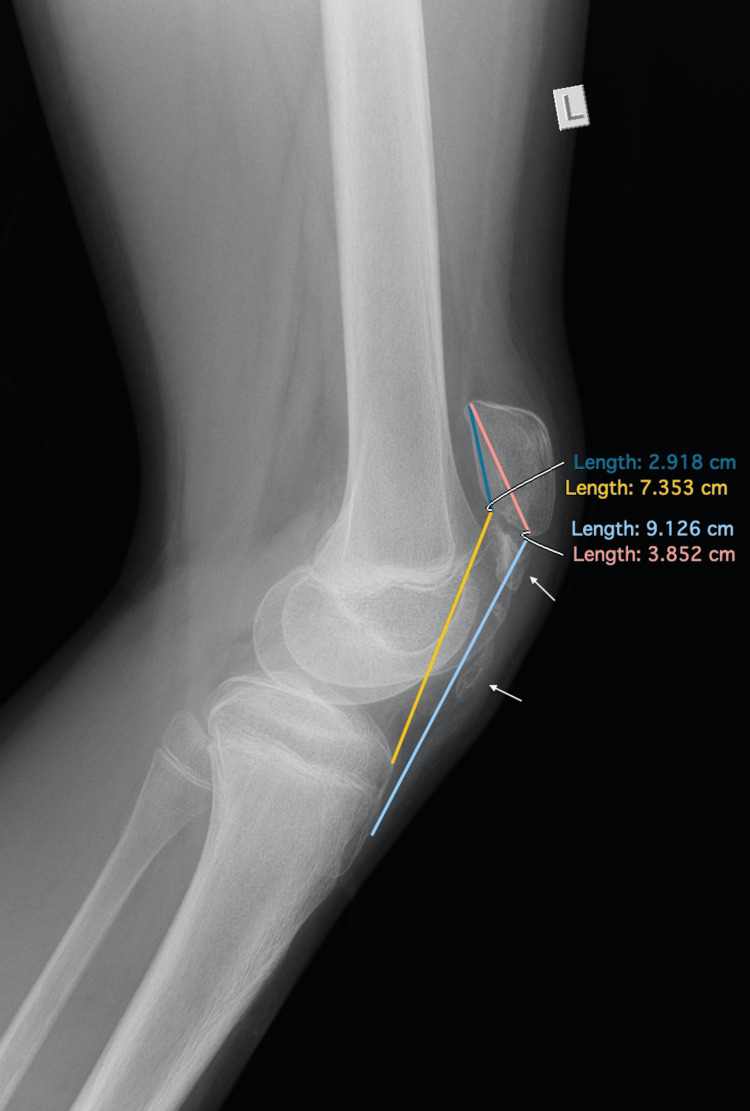
A preoperative X-ray of the left knee taken four months after the injury revealed proximal patellar tendon ossification (white arrows) with an Insall-Salvati ratio of 2.36 and a Caton-Deschamps index of 2.52.

**Figure 2:**
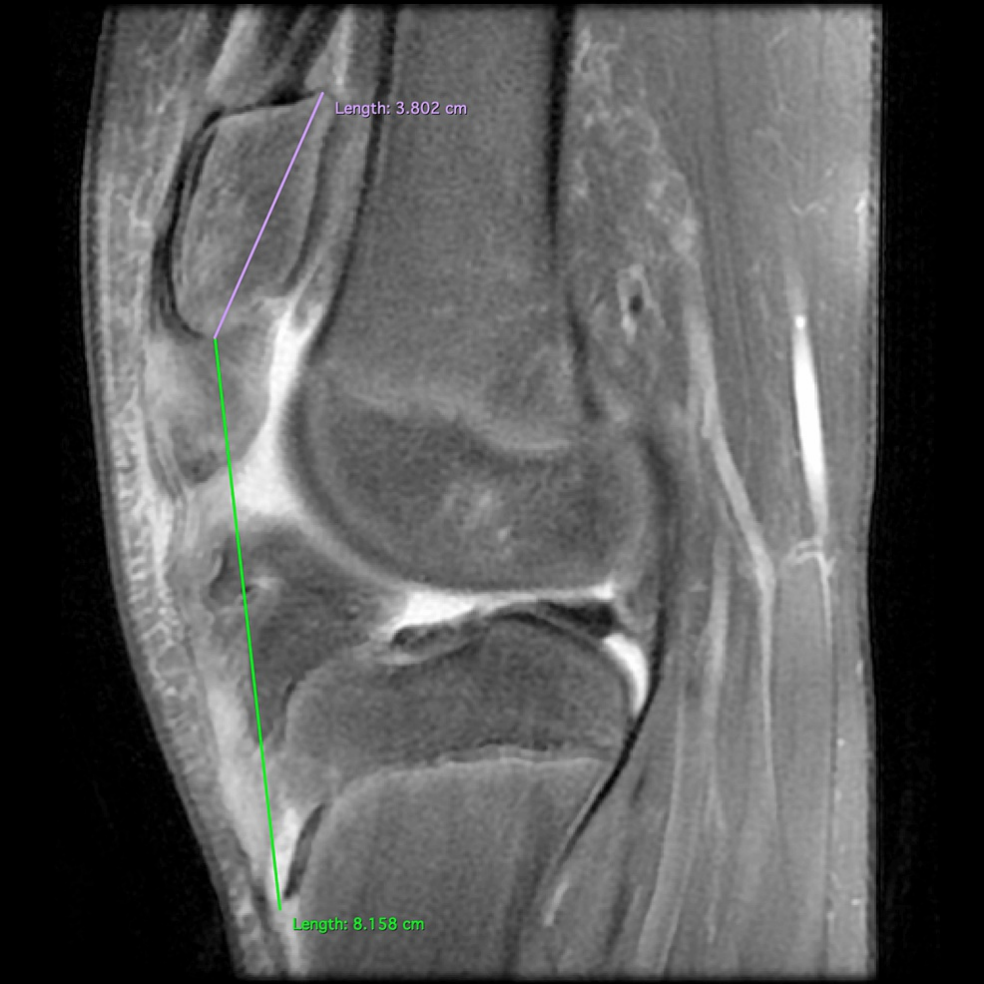
Sagittal view of the left knee MRI demonstrating proximal patella migration.

Physical examination revealed diffuse swelling over the anterior knee, tenderness on palpation, and a palpable gap below the inferior pole of the patella. No signs of neurovascular compromise or inflammation were observed. Interestingly, the patella appeared to be remarkably proximally translated. The radiological examination revealed an open physis with no obvious avulsion fracture but an exceptional proximal migration of the patella with both the Insall-Salvati ratio and the Caton-Deschamps index calculated > 2 (Figure 1). In addition, ossification of the patellar tendon close to the distal patellar pole was observed (Figure 1). The MRI demonstrated a patella alta as well, with a mid-to-proximal intrasubstance stretch injury of the tendon (Figure 2). No meniscal or other intra-articular lesion was demonstrated in the MRI examination.

**Figure 3:**
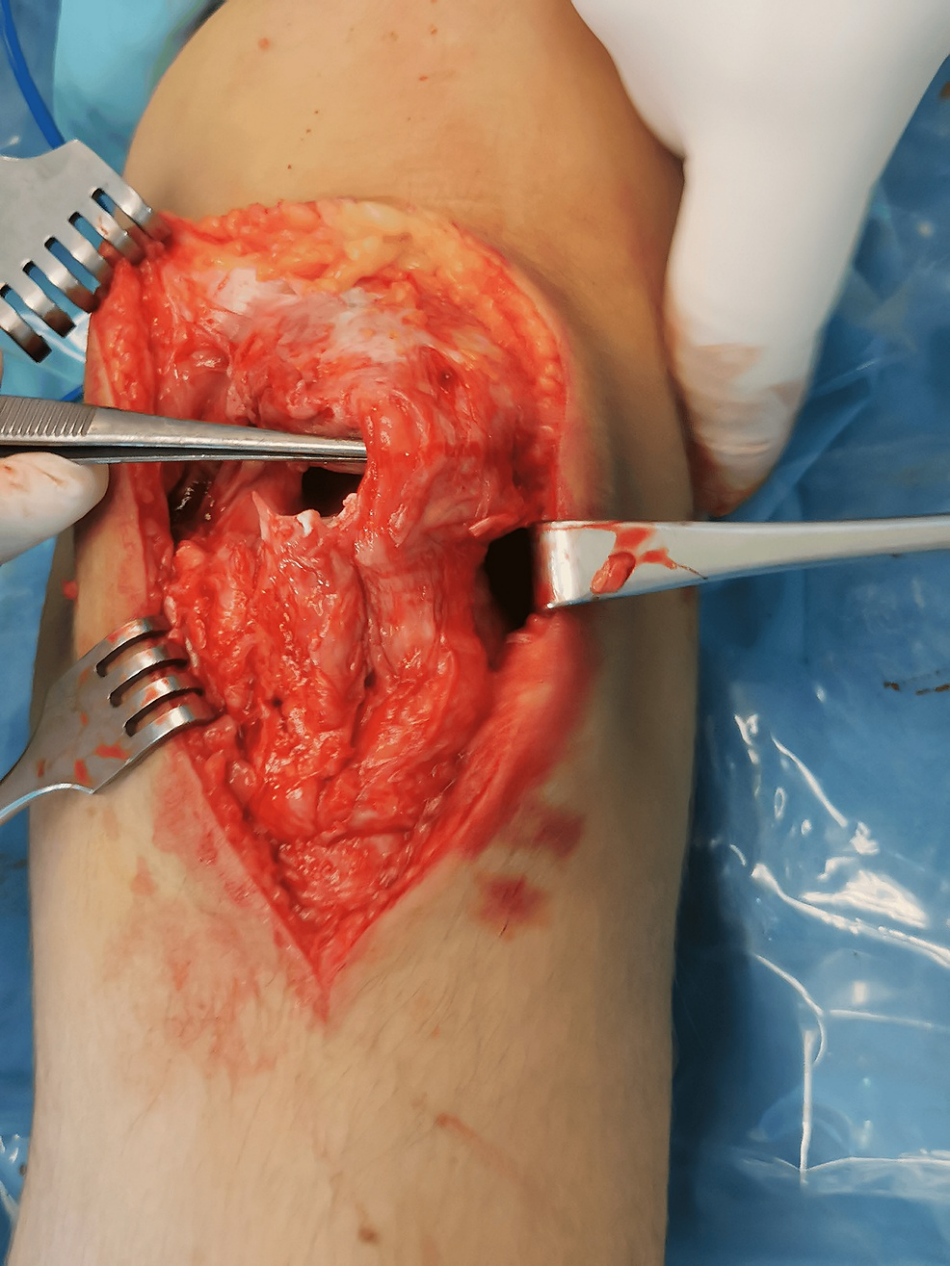
Tendon defect after ossified segment excision.

**Figure 4:**
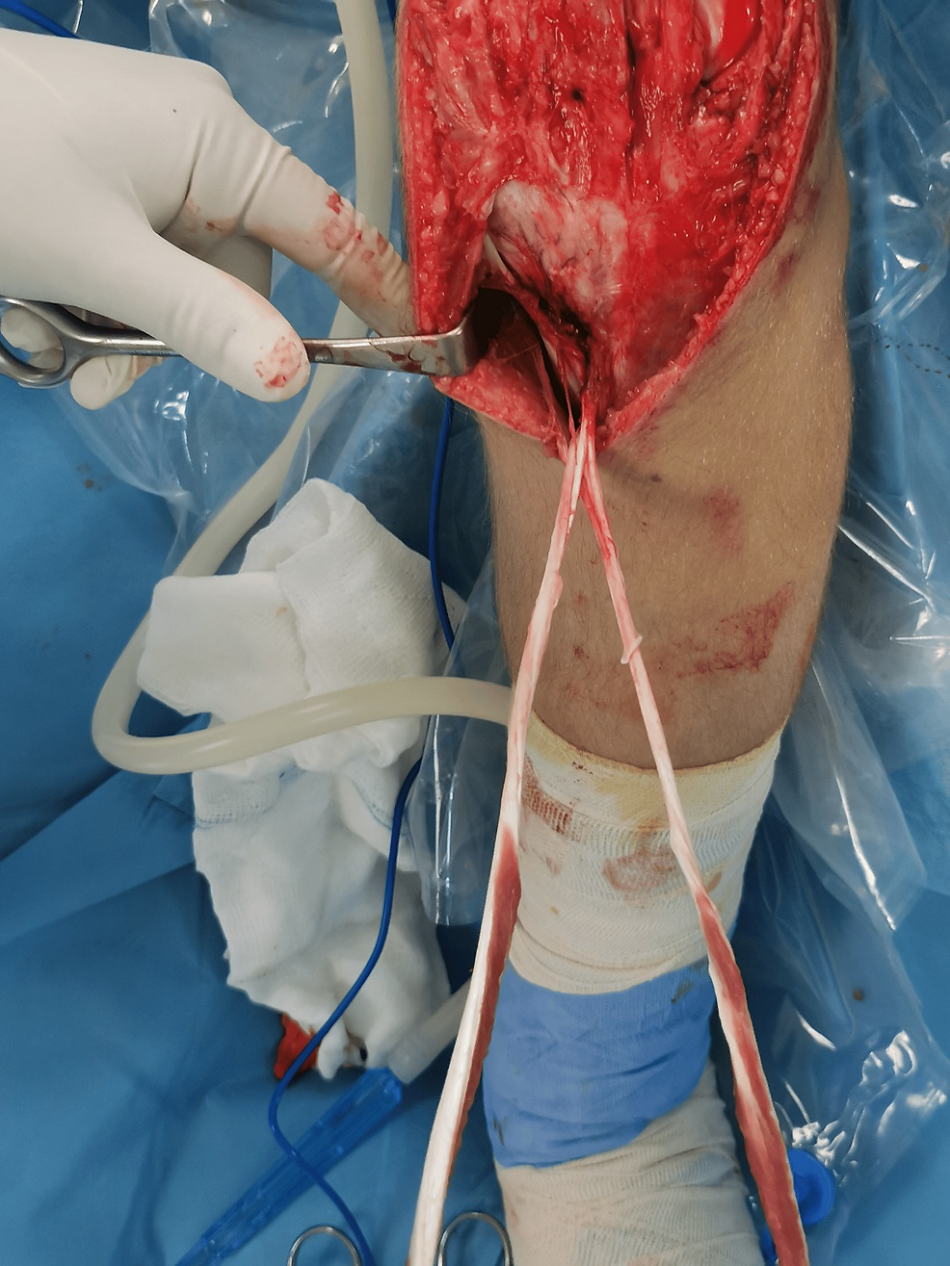
Semitendinosus and gracilis tendons were harvested with an open hamstring tendon stripping device, leaving them attached distally.

**Figure 5:**
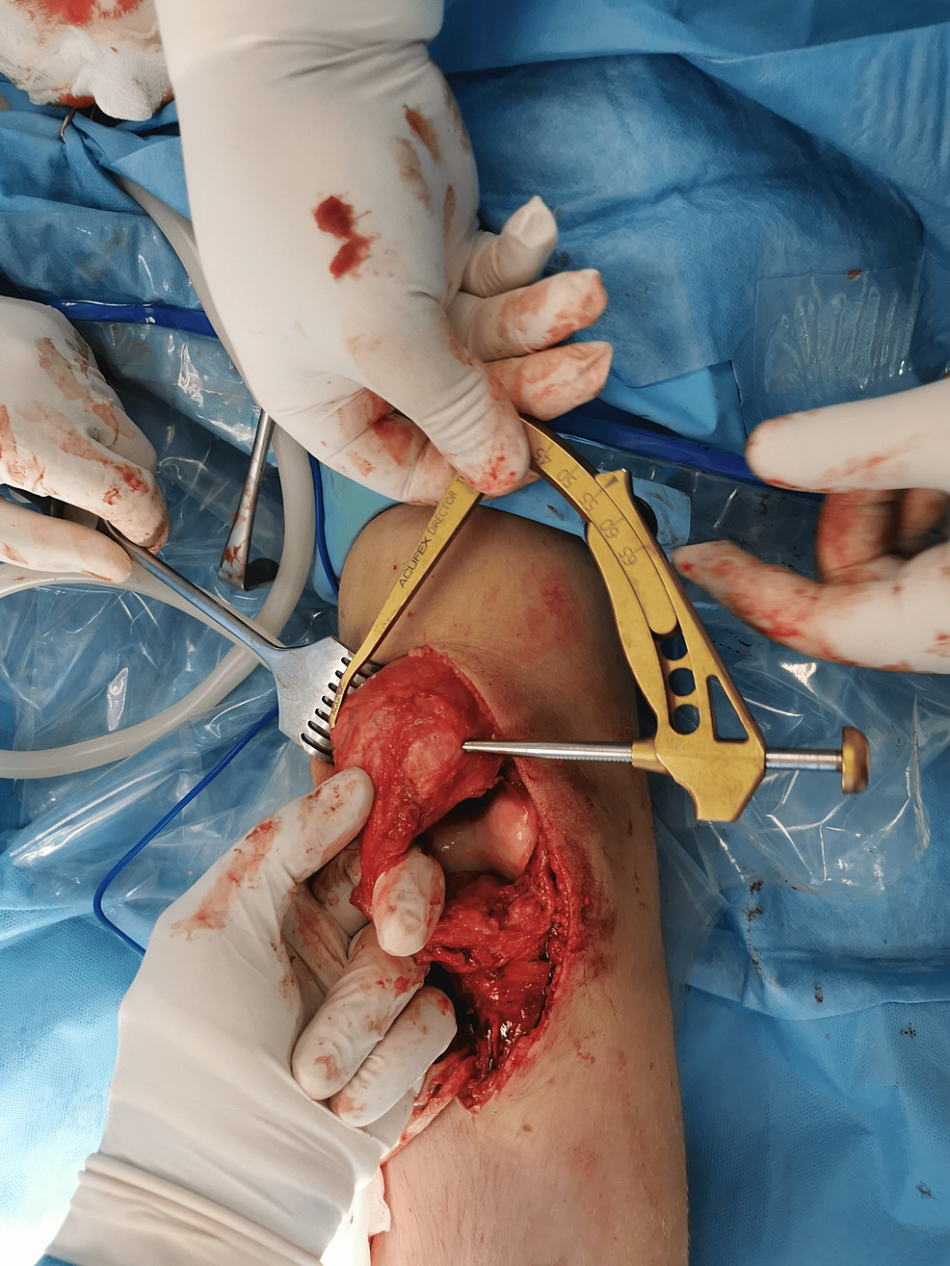
The anterior cruciate ligament tibial guide is placed in the mid-portion of the patella.

**Figure 6:**
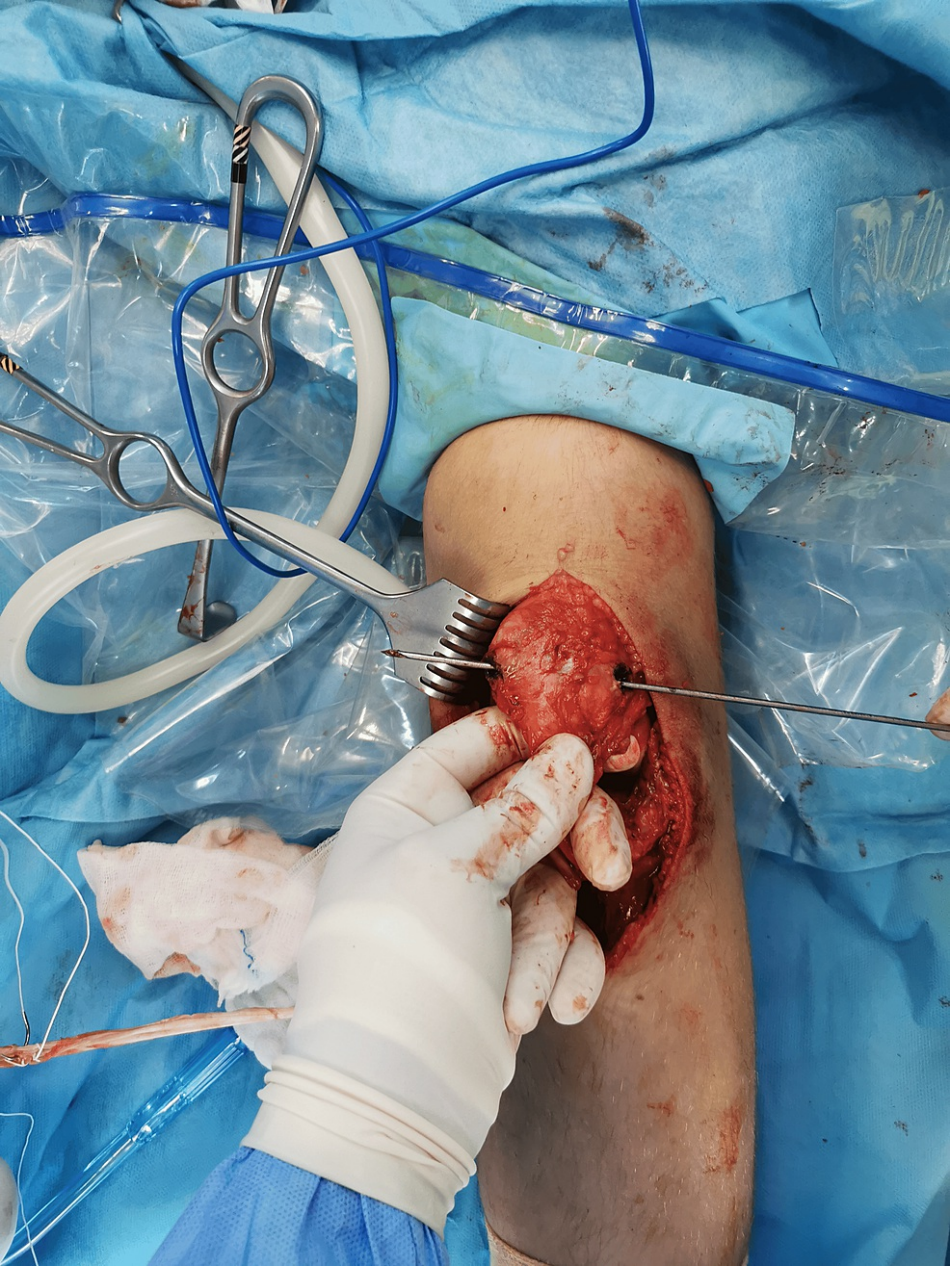
A horizontal eyelet pin was inserted from lateral to medial, and the tunnel was over-drilled with a 5 mm reamer.

**Figure 7:**
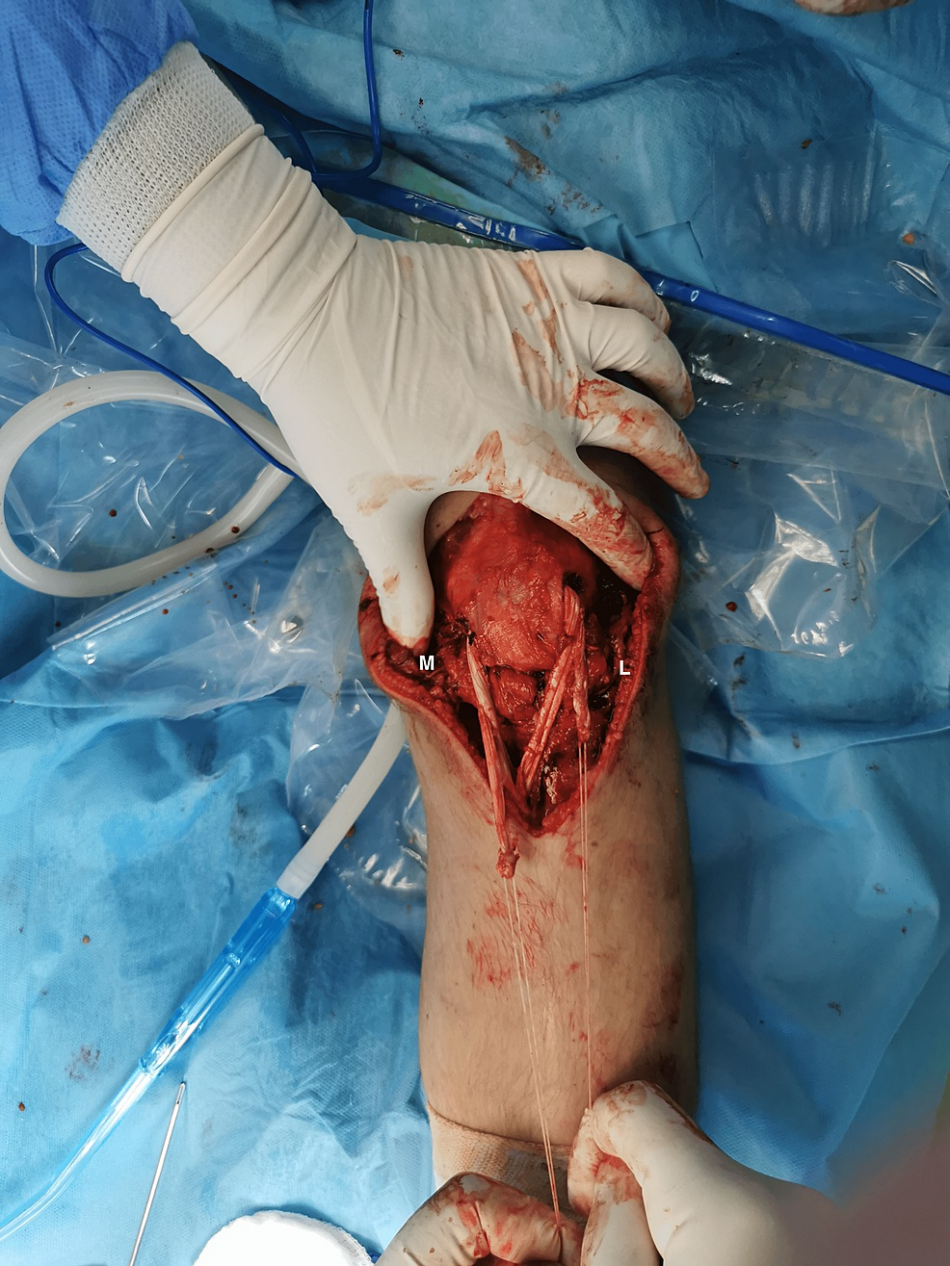
The semitendinosus tendon was pulled from L to M, and the gracilis tendon from M to L through the patella tunnel using a passing stitch. M: medial; L: lateral.

Surgical treatment was proposed, which was finally performed four months after the initial trauma. The patient was placed in a supine position, general anesthesia was induced, and a padded tourniquet was placed on the upper thigh. A longitudinal midline incision was made over the anterior aspect of the knee, and the tendon defect was exposed. Excision of the ossified segments, just below the distal pole of the patella, was initially performed (Figure 3). STG tendons were harvested and left attached distally (Figure 4). The ends of each tendon were whipstitched with No. 2 nonabsorbable sutures to expedite graft passage. Afterward, with the knee in extension, an ACL guide was placed in the midportion of the patella (Figure 5), and a transverse eyelet pin was drilled from lateral to medial (Figure 6). The tunnel was over-drilled with a 5 mm Endobutton reamer, and the semitendinosus tendon was pulled from lateral to medial and the gracilis tendon from medial to lateral through the patella using a passing switch (Figure 7). At this point, the patellar tendon was detached from its insertion on the tibial tubercle due to frayed tissues in this zone and not enough tension in the tendon.

**Figure 8:**
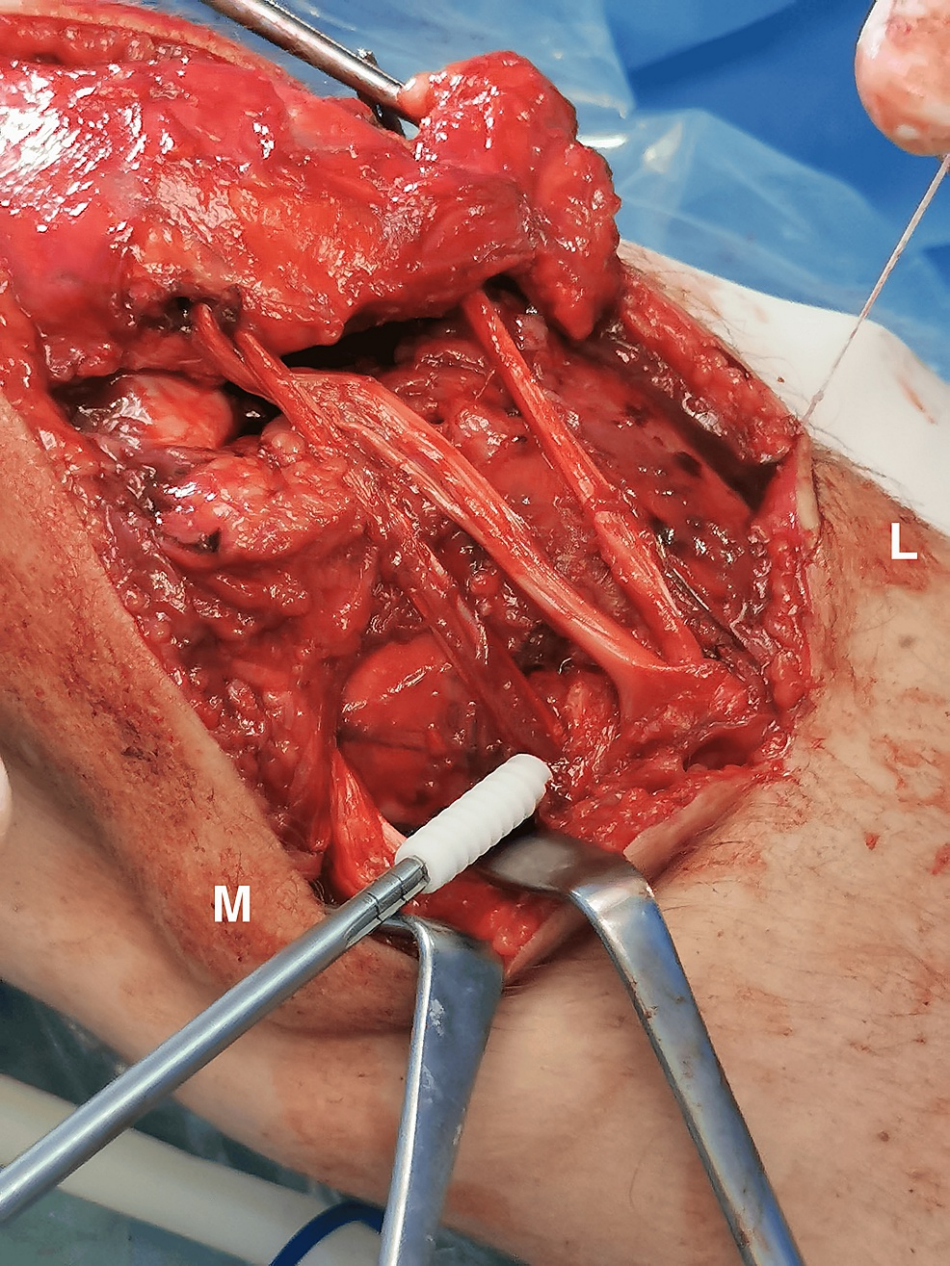
Reposition of the patella to its correct height and semitendinosus and gracilis tendons fixation into the tibial tunnel with a 7 x 30 mm bioabsorbable screw. M: medial; L: lateral.

**Figure 9:**
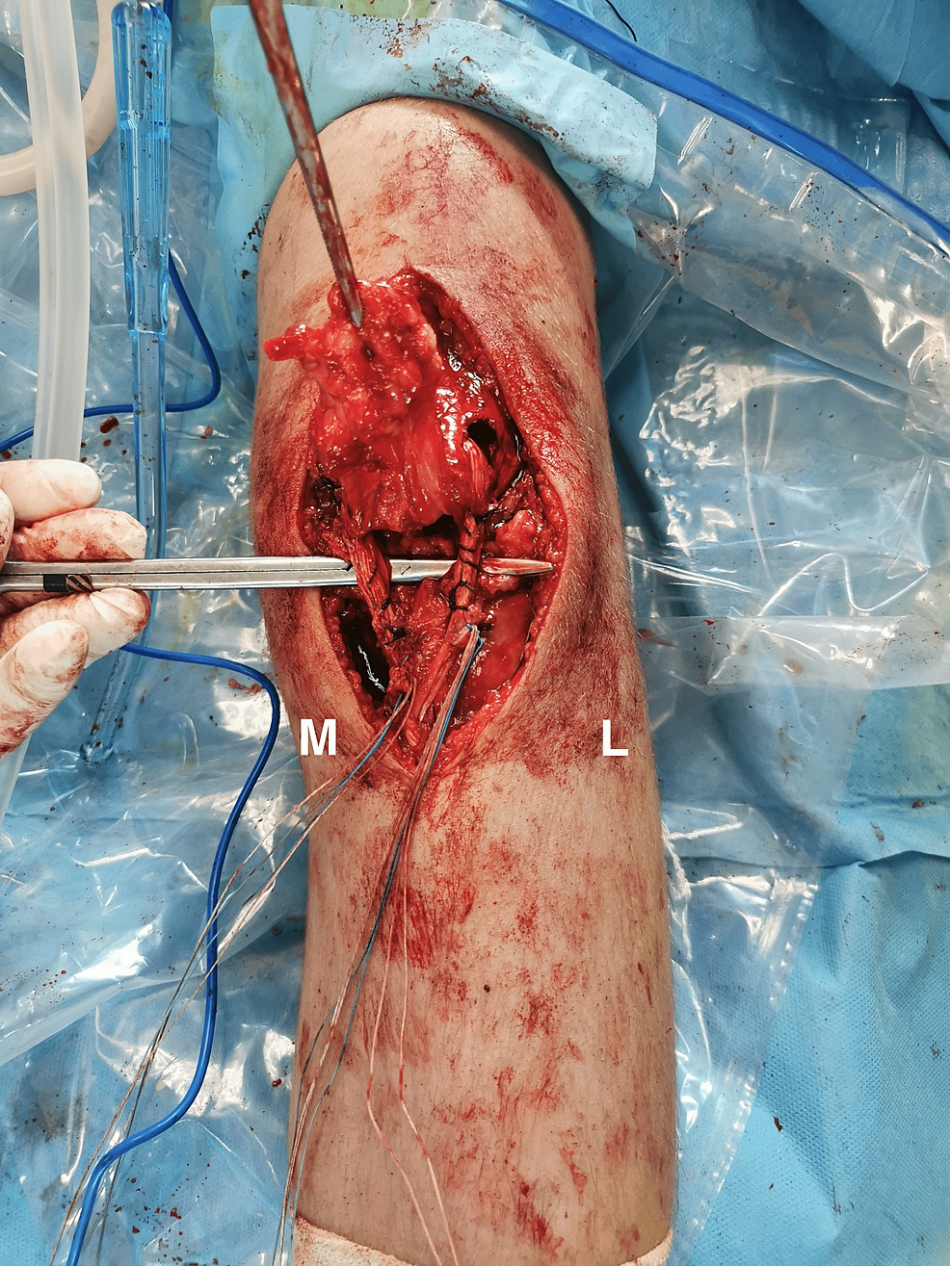
Semitendinosus and gracilis tendons sutured together on both sides. M: medial; L: lateral.

**Figure 10:**
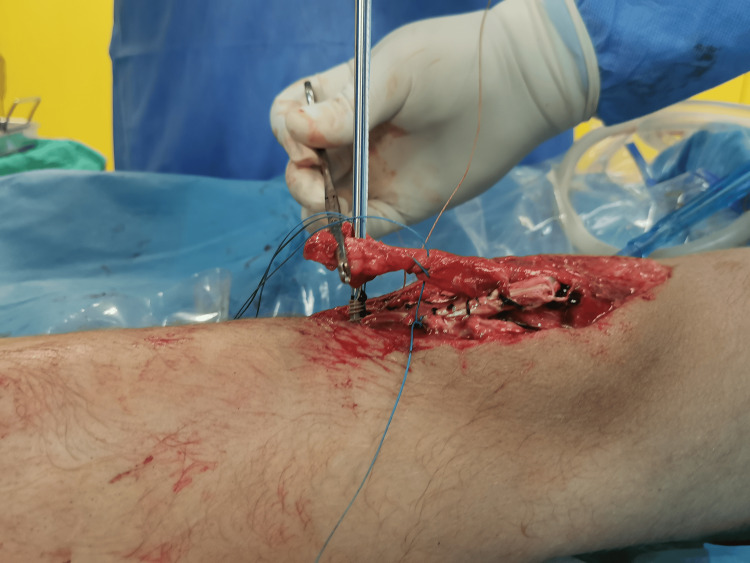
Repositioning of the patellar tendon to its insertion under the proper tension and securing it with the first of the two PEEK suture anchors (5.5 mm).

**Figure 11:**
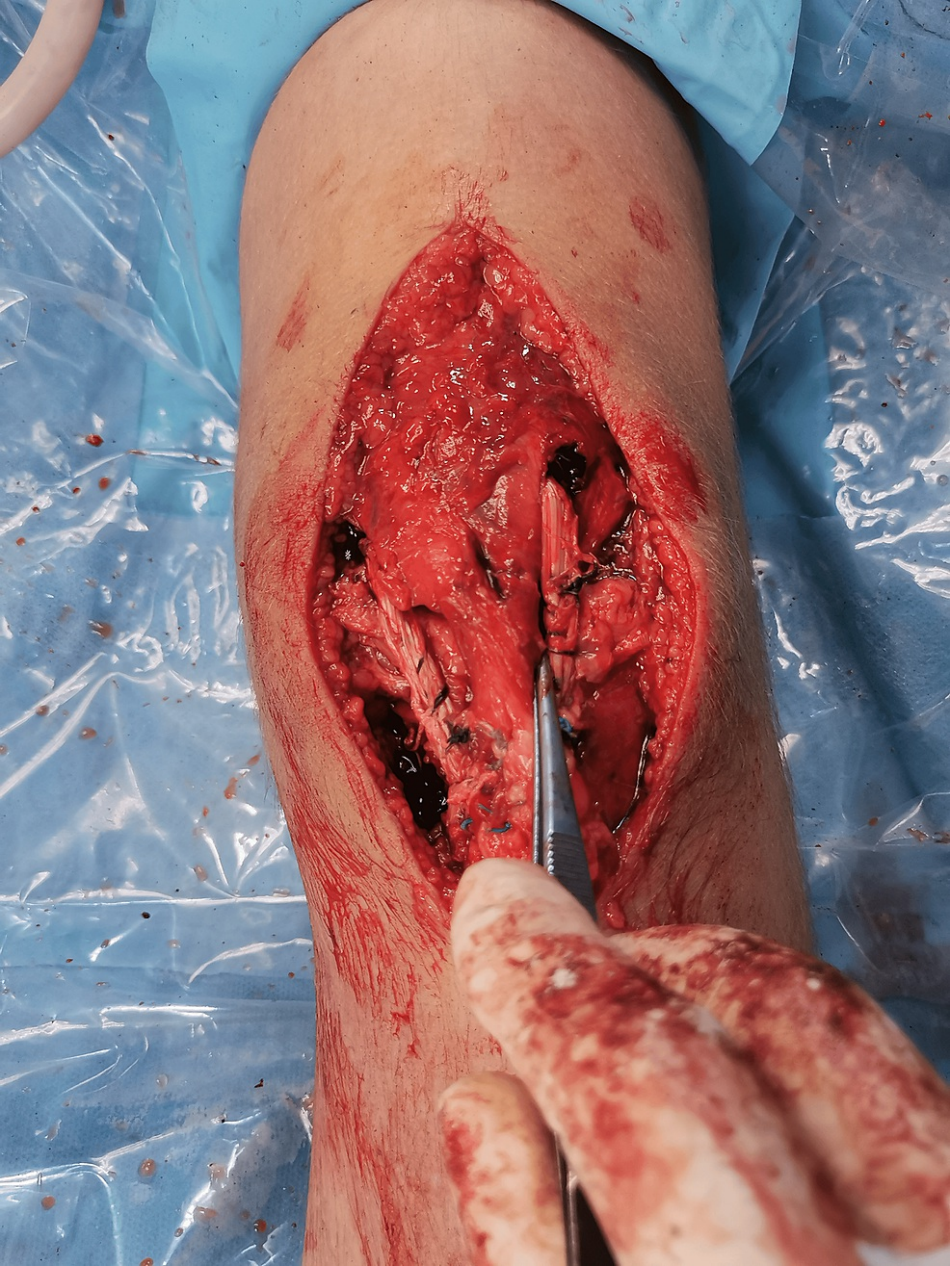
The final result of the reconstruction.

The medial and lateral aspects of the tibia were exposed, and once again, a similar tunnel was drilled with the use of the ACL guide, distal to the open physis. After the repositioning of the patella to its correct height, defined as the level where the lower pole is at the roof of the intercondylar notch, the semitendinosus tendon was pulled from medial to lateral through the tibial tunnel, while the gracilis tendon was pulled from lateral to medial. The tendons were fixed into the tibial tunnel with a 7 mm x 30 mm bioabsorbable screw (Figure 8). Nonabsorbable sutures were also used to suture STG tendons to one another on both sides in their whole length, from the patella to the tibial tunnel, to reinforce the fixation (Figure 9). Moreover, the patellar tendon was repositioned to its insertion at the tibial tubercle under the proper tension and was secured with two PEEK (5.5 mm) suture anchors (Figure 10). At this point, the final reconstruction was evaluated by moving the knee from 0 to 90 degrees of flexion. No patella maltracking or increased graft tension was noted (Figure 11).

Postoperatively, the knee was immobilized in a brace, locked in an extension, and partial weight-bearing was instructed with the support of crutches. Flexion to 30° was allowed in the first two weeks, and from the third postoperative week, 15° was added each week with a goal of 90° of flexion at six weeks. Physical therapy was encouraged from the first postoperative day to prevent stiffness, control edema, and minimize quadriceps dysfunction. After the sixth week, the brace was discontinued, knee flexion was advanced beyond 90°, and progressively full weight-bearing (FWB) was allowed until the third postoperative month.

**Figure 12:**
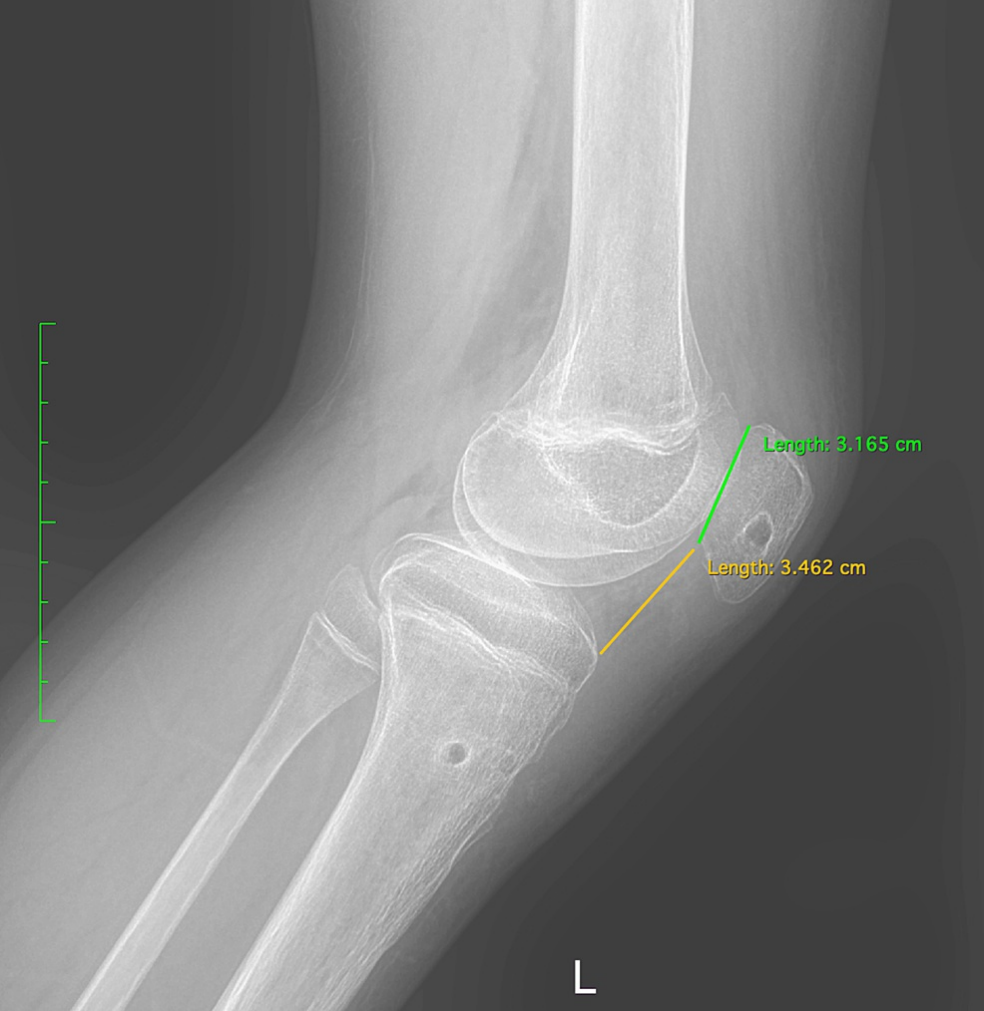
Postoperative X-ray of the left knee after one year, with normal Caton-Deschamps index.

The patient was evaluated radiographically and functionally by knee assessment scores, including Kujala, International Knee Documentation Committee (IKDC), and Lysholm, at zero, three, six, and 12 months postoperatively. At one-year follow-up, the Kujala score had increased from 13% to 95%, the IKDC score from 2.3% to 93.1%, and the Lysholm score from 6.3% to 99%. The Caton-Deschamps index was 1.09 (Figure 12). From the third postoperative month, the patient had a full range and painless motion (0°-135°) and was walking with FWB with a normal gait (Video 2). The postoperative period was uneventful, without complications, and the patient returned to his previous activity.

**Video 2:** Postoperative knee range of motion at three months.

## Discussion

Tendon rupture is more common in patients with systemic diseases such as diabetes mellitus, chronic renal disease, systemic lupus erythematosus, and steroid medication [8]. However, our patient was otherwise healthy, and the injury was due to a traumatic event.

The available surgical techniques for chronic patellar tendon tears are extremely challenging and demanding, with unpredictable outcomes [9]. The surgeon should consider how to deal with potential poor tissue quality, the formation of adhesions, muscle contractures, stiffness, and patella retraction. The latter situation may demand a quadriceps lengthening technique, such as quadriceps tendon V-Y or Z plasty, taking into account quadriceps muscle atrophy as well. However, kids seem to form fewer adhesions and stiffness than adults.

A recent systematic review showed that while there is no one best technique or type of graft, all of them can yield good results, including improved strength, nearly excellent postoperative functional scores, and a low rate of complications [10]. The use of contralateral bone-patellar tendon-bone (BPTB) is related to high donor-site morbidity, anterior knee pain, loss of sensitivity, or even difficulty in kneeling [11]. However, those in favor of this graft type argue that with this method, a precise reconstruction of the extensor mechanism can be achieved regarding patella height with a solid fixation [9,12]. Besides these, BPTB autograft should be excluded in skeletally immature patients because of the high risk of harming the physes.

Allografts such as BPTB or Achilles tendon are widely used in young and active people [13-15], but in children, only one case is reported as an augmentation technique for the repair of the patellar tendon using an Achilles tendon allograft without a calcaneal bone block [5]. Allografts, despite their high cost, are a viable option for restoring extensor function and range of motion, particularly in cases where the remaining tissues are absent or severely diminished. However, a recent systematic review and meta-analysis revealed that allografts have a significantly higher failure rate than autografts in patients under 19 years old who underwent ACL reconstruction with a minimum of two years of follow-up [16]. Comparable outcomes have been published for synthetic grafts, such as ligament augmentation reconstruction system (LARS) ligament [17].

In our case, we performed a reconstruction of the patellar tendon rupture using hamstring tendons with preserved insertions, without the need for synthetic materials. It has been described as ideal for preserving its viability and the revascularization required to enhance healing [18]. STG tendons are rich in tendon fibers and can be a strong graft, as the double semitendinosus tendon strand showed a maximum average load of 3395N, whereas the double gracilis tendon strand showed 2573N, respectively. This means that these double tendon strands are stronger than the BPTB strand, with a maximum average load of 3855N [19]. In addition to this reconstruction, we repositioned the remnant tissue of the native patellar tendon for its insertion under the proper tension.

By conducting a review of the literature, only six articles were found regarding patients with immature skeletons, confirming the rarity of this condition and the value of case reports on such issues [3-7]. Special consideration must be given to open physes during the procedure. The employed anchors must not interfere with the epiphysis, and fluoroscopic guidance should be used if the surgeon is in doubt.

Potential complications include patella fracture, when the transosseous tunnel is made, and persistent knee pain, which is reported to be the most common one [10]. Other challenges that a surgeon might encounter include decreased quadriceps strength, persistent hemarthrosis, and wound complications.

## Conclusions

Our case study has demonstrated that patellar tendon rupture can be repaired with a hamstring autograft in adolescents, resulting in complete strength, range of motion, and a pain-free gait. The technique described is reproducible, by using two anchors and one bioabsorbable screw through the transtibial tunnel and is a valuable addition to the therapeutic quiver in the treatment of this type of injury. Furthermore, there is no need for expensive allografts, and harming the physis is carefully avoided. Such case reports provide a significant contribution to the currently limited research. The main limitation is that these results are short-term, and to firmly conclude a decreased risk of re-rupture or other late complications, a long-term follow-up is required.
